# The Effect of Superstructures Connected to Implants with Different Surface Properties on the Surrounding Bone

**DOI:** 10.3390/jfb6030623

**Published:** 2015-07-24

**Authors:** Katsunori Koretake, Hiroshi Oue, Shinsuke Okada, Yosuke Takeda, Kazuya Doi, Yasumasa Akagawa, Kazuhiro Tsuga

**Affiliations:** 1 Department of Advanced Prosthodontics, Institute of Biomedical & Health Sciences, Hiroshima University, 1-2-3 Kasumi Minami-ku, Hiroshima 734-8553, Japan; E-Mails: katsu@hiroshima-u.ac.jp (K.K.); shinsuke0517@hiroshima-u.ac.jp (S.O.); y-takeda0117@hiroshima-u.ac.jp (Y.T.); kazuya17@hiroshima-u.ac.jp (K.D.); tsuga@hiroshima-u.ac.jp (K.T.); 2 Department of Prosthetic Dentistry, School of Dentistry, Ohu University, 31-1, Tomitamachi-Aza Misumidou, Koriyama 963-8611, Japan; E-Mail: akagawa@den.ohu-u.ac.jp

**Keywords:** implant, superstructure, removal torque, remodeling, marginal bone loss

## Abstract

The objective of this study was to investigate how the connection of superstructures to implants with different surface properties affects the surrounding bone. The right and left mandibular premolars and molars of 5 dogs were extracted. After 12 weeks, a machined implant was placed mesially and an anodized implant was placed distally on one side of the edentulous jaw, with the positions reversed on the opposite side. Twelve weeks after implantation, splinted superstructures were set to the implants. At 24 weeks after implantation, the implant stability quotient (ISQ) was measured, radiographs were obtained. Removal torque values were measured and histologic observation was performed. The ISQ values at 24 weeks after implantation were not significantly different between the groups. The removal torque values were significantly different between the distal anodized and distal machined implants (*p* < 0.05). From 12 to 24 weeks, marginal bone losses were not significantly different between the groups. Fluorescent observation of tissue samples revealed bone-remodeling activity around all of the implants. The results of this study suggest that when implants with different surface properties are connected, machined implants at the most distal sites might be a potential risk factor for implant-bone binding.

## 1. Introduction

The earliest reports focused on prosthetic devices for edentulous patients that were supported by implants [1]; however, in recent years, implants have also been indicated for defect prostheses in partially edentulous patients [2]. For implant treatment of partially edentulous patients, the remaining teeth and the implant require different support mechanisms, and, therefore, we considered that the jawbone could be subjected to more complex stressors compared to patients with a bone-anchored full bridge.

Reports on the success of fixed bridges for implants show variable results. In a prospective cohort study of 77 partially edentulous patients, Wyatt *et al*. [3] reported a 94% success rate of implant-supported fixed prostheses, and 97% of the prostheses were stable. In a prospective cohort study of 250 patients with 106 independent crowns, 42 partial cantilever fixed prostheses, and 137 partial fixed prostheses (five with bone-anchored full bridges and 13 with implant/tooth-supported prostheses and implant overdenture treatments), there was no difference in the 7-year success rate between the groups, and there was no influence of maxillary or mandibular location [4]. In contrast, Bavbek *et al*. [5] reported that peri-implant stress increased with the use of a mesiodistal implant/tooth-supported connected prosthesis. Moreover, Zarone *et al*. [6] used three-dimensional finite element analysis to design six different kinds of superstructures for mandibular edentulous sites, and reported that although implant connection could produce natural biomechanical behaviors, significant stress was generated in more distally located implants and superstructures of the symphysis menti area.

Recently, various types of implants with different surface properties have been rapidly developed with the aim of achieving early osseointegration [7]. This is critical to increase the success rate of implant treatments, particularly as older implant types are gradually being phased out. Thus, even if a new defect site occurs several years after implantation and additional implantation becomes necessary, use of the same type of implant may not be possible. For example, the use of a machined implant surface was common in the past several decades and the use of rough-surface implants has become increasingly common in recent years. However, there is no evidence to show whether connecting superstructures to implants with different surface properties could affect outcomes. In view of these circumstances, we designed the present study to investigate the influence of connecting superstructures to implants with different surface properties on the surrounding bone.

## 2. Results and Discussion

### 2.1. General Characteristics of Implants

None of the implants showed mobility or loss. No inflammation was observed in the peri-implant mucosa and gingiva.

### 2.2. Implant Stability Quotient (ISQ) Values

**Figure 1:**
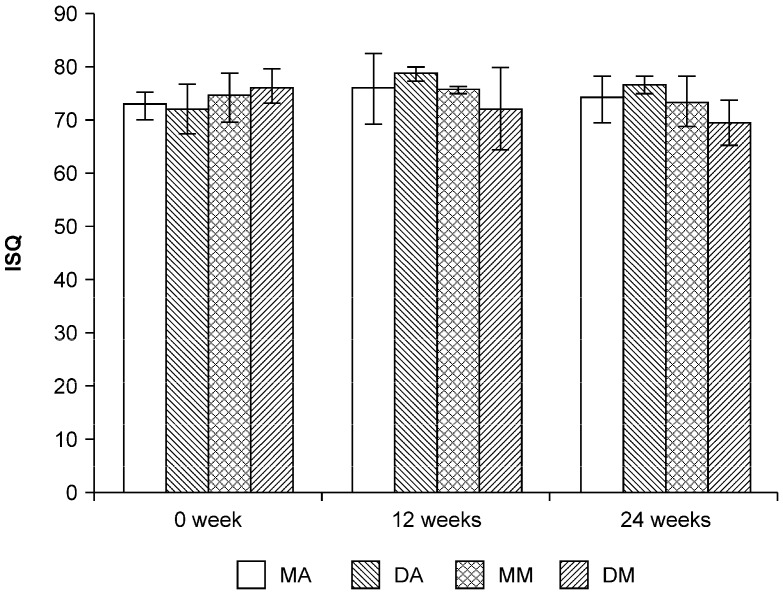
Implant stability quotients (ISQs) in the different implants. There were no significant difference in ISQ values among the groups or weeks. MA: mesial anodized implant; DA: distal anodized implant; MM: mesial machined implant; DM: distal machined implant.

Figure 1 shows the implant stability quotient (ISQ) values of each implant at 0, 12, and 24 weeks. All implants showed high ISQ values. At 0 weeks, ISQ values of mesial anordized (MA), distal anordized (DA), mesial machined (MM), distal machined (DM) were 72.7 ± 2.5, 72.0 ± 4.6, 74.3 ± 4.5, 76.2 ± 3.3, respectively. At 12 weeks, ISQ values of MA, DA, MM, DM were 75.8 ± 6.8, 78.7 ± 1.3, 75.3 ± 0.6, 72.3 ± 7.8. At 24 weeks, ISQ vales of MA, DA, MM, DM were 73.9 ± 4.4, 76.5 ± 1.7, 73.5 ± 4.8, 69.3 ± 4.2. There were no significant difference in ISQ values between implants at any time period (*p* > 0.05).

### 2.3. Removal Torque Values

**Figure 2:**
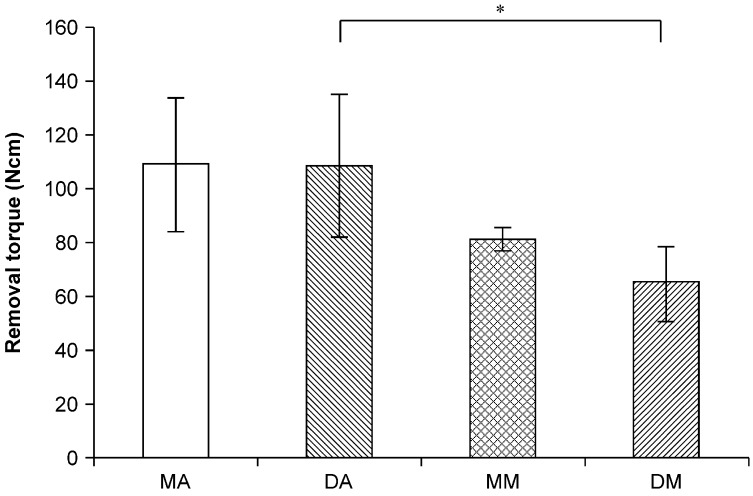
Removal torque in the different implants. A significant difference was observed between the mesial and distal anodized and the distal machined implants (*p* < 0.05). MA: mesial anodized implant; DA: distal anodized implant; MM: mesial machined implant; DM: distal machined implant.

Figure 2 illustrates the 24-week removal torque values. The removal torque values of the mesial anodized, distal anodized, mesial machined, and distal machined groups at 24 weeks were 108.5 ± 25.2, 107.7 ± 26.6, 80.9 ± 4.4, and 64.7 ± 13.8 Ncm, respectively. A significant difference was observed between the distal anodized and machined implants (*p* < 0.05).

### 2.4. X-ray Observations

**Figure 3:**
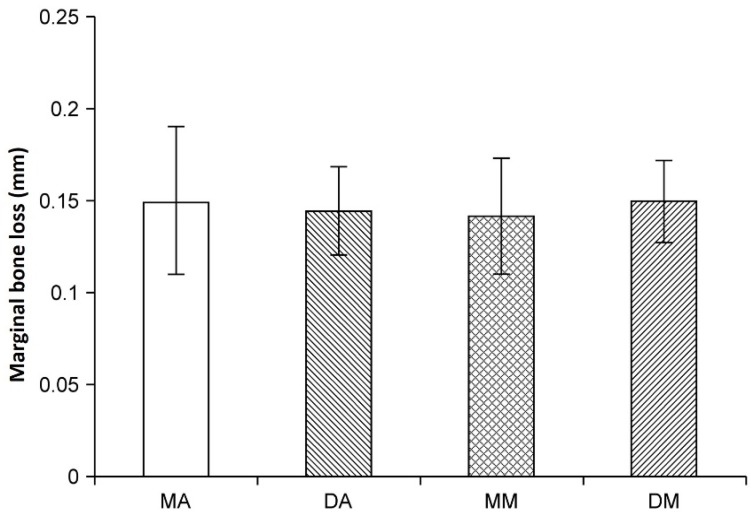
Vertical marginal bone loss (mm) measured on X-ray radiographs. There were no significant differences between the groups. MA: mesial anodized implant; DA: distal anodized implant; MM: mesial machined implant; DM: distal machined implant

Figure 3 illustrates the changes in MBL with each implant type. The change in MBL from 12 to 24 weeks for the mesial anodized, distal anodized, mesial machined, and distal machined implants was 0.15 ± 0.041, 0.15 ± 0.025, 0.14 ± 0.032, and 0.15 ± 0.020 mm, respectively. Thus, the MBL for all groups was within 0.2 mm, and no significant difference was observed between the groups (*p* > 0.05).

### 2.5. Histological Observation

**Figure 4:**
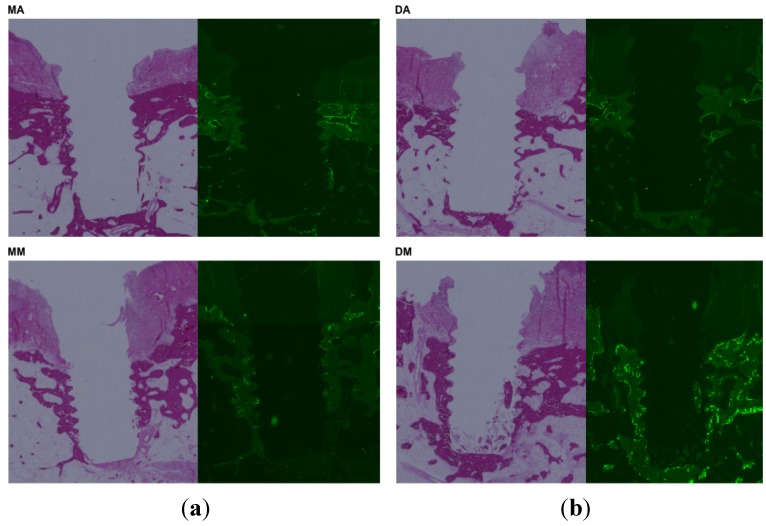
Histological observations of bone specimens: (**a**) light microscopy; (**b**) fluorescence microscopy. DM specimens produced the strongest fluorescent signal. MA: mesial anodized implant; DA: distal anodized implant; MM: mesial machined implant; DM: distal machined implant.

Representative histological images are shown in Figure 4. Inflammation was not observed around any of the implants, and there was no involvement of soft tissue noted. Under fluorescence microscopy, mild bone remodeling activity was observed around the mesial anodized, distal anodized, and mesial machined implants, whereas strong remodeling activity was observed around the distal machined implants.

### 2.6. Discussion

Implant therapy is a common treatment to replace missing teeth. When implants were first introduced, machined implants were the standard [1,2,3,4,5,6,7,8,9]. Since then, various types of implants with different surface properties have been developed with the objective of earlier attainment of osseointegration. Since these new implants are rapidly replacing machined implants, an implant placed at a new defect site several years after the first implant might have different surface properties. We tested the hypothesis that connecting implants with different surface properties might increase the stress on the surrounding bone and trigger bone resorption. In fact, if we hypothesize the clinical situation, implant placement of anodized implant should have been performed several years after machined implant placement. However, if machined implant placement were performed several years before anodized implant placement, surrounding bone condition of both implants may be different. In particular, it is considered that bone level could be different between implants or there could exist inflammation around machined implant. To clarify the potential effect of connecting implant with different surface properties, we considered that the placement of different implant should be done simultaneously.

There are various methods for assessing the stability of the bone-implant interface [10,11]. In this study, we measured the removal torque value for the implant bodies, along with the ISQ using a resonance frequency analyzer. The ISQ values were not affected by the connection of implants with different surface properties at any time point. However, the removal torque values of the distal machined implants were significantly lower than those of the mesial anodized and distal anodized implants.

Very little is known about the correlations between ISQ values and removal torque values; however, ISQ values are thought to indicate implant stability and removal torque values are considered to indicate the bone–implant bonding strength. Degidi *et al*. [12] concluded that the relationship of resonance frequency analysis and bone structure is not fully understood. Cordioli *et al*. [13] measured the bone-to-implant contact rate (BIC) and removal torque values for implants with four different surface properties (machined surfaces, grit-blasted surfaces, plasma-spray surfaces, and anodized surfaces), and reported that implants with anodized surfaces exhibited higher values of BIC and removal torque than the other implants. Rismanchian *et al*. [14] also compared the BIC and removal torque values for XiVe and Nisastan implants which were similar with surface characteristics (both implants have an acid-etched and blasted surface) and reported that XiVe implants exhibited significantly higher values of removal torque. However, BIC values in both Xive and Nisastan implants were almost equal. The authors suggested that these results reflect limited differences in the acid treatments or spray methods used for the manufacturing of these two implants, which triggered different biological reactions in the bone surrounding the implants. Similarly, our results suggest that implants with machined and anodized surfaces triggered significantly different biological reactions. It is considered that the strength of bone and implant connection of anodized implant is higher than that of machined implant. We considered that the connection of anodized and machined implant supported prosthesis might approach the condition of implant/tooth connection. Therefore, connecting different surface implant supported prosthesis may amplify the stress to the bone surrounding the machined implants.

Furthermore, the flexure of bone during mandibular function has been discussed in various publications investigating peri-implant success. Al-Sukhun *et al*. [15] and Abdel *et al*. [16] have suggested that momentary flexure occurs simultaneously with mandible function. Zarone *et al*. [6] demonstrated that substantial stress was caused by flexure of the bone applied to implant-supported prostheses during mandibular function. These findings suggest that although the periodontal ligament corrects for the torsional stress caused by connections with natural teeth, with long-lasting strain, the superstructural connection of implants could potentially damage the bone-implant interface.

In addition to the stress caused by superstructural connections, a significantly higher amount of stress has been reported on the bone around the more distally located implants and implants of the symphysis menti area than bone at other areas. In their review, Law *et al*. [17] stated that mandibular flexure is a multifactorial phenomenon impacted by bone quality, bone mass, the number and position of implants, the method of impression, and the prosthesis design. Given that much remains unclear about the importance of flexure of the mandibular bone in cases of successful implants, this factor should be taken into account when designing a prosthesis.

Histologically, stronger bone remodeling activity was noted around the distal machined implants compared to the other implants evaluated. However, no difference in the amount of bone resorption was observed on radiography. This phenomenon could occur if the loss of osseointegration was preceded by active bone remodeling [18]. Therefore, bone resorption might be expected to occur in the next several months.

One limitation of the present study is the relatively small sample size. Nonetheless, the limited results of our study suggest that when implants with different surface properties are mesially connected for treating partial edentulism, the most distal implants with mechanically polished surfaces (*i.e.*, machined implants) need to be carefully monitored.

## 3. Experimental Section

### 3.1. Ethics Statement

The animal research protocol was conducted in accordance with the current version of the Japan Law on the Protection of Animals. This study was approved by the Research Facilities Committee for Laboratory Animal Science at the Hiroshima University School of Medicine, Hiroshima, Japan. All surgery was performed under general anesthesia, and all efforts were made to minimize suffering during the experimental period.

### 3.2. Study Design

**Figure 5:**
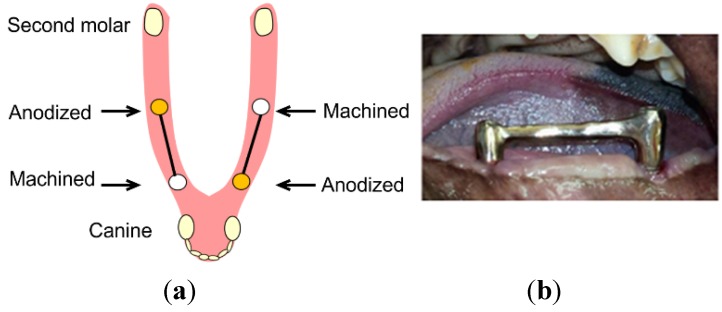
Implant configuration (**a**); Different implants were connected by a superstructure (**b**).

We considered that *in vivo* study would be most appropriate to clarify the dynamics of peri-implant bone. Five 2-year-old male Beagle-Labrador mixed-breed dogs were used as the experimental animals. The small molars (P1–P4) and molars (M1) on both mandibles were extracted. After a 12-week healing period, implant placement cavities were formed according to manufacturer-recommended protocols. The surgical procedures were performed under general anesthesia with sodium pentobarbital (30 mg/kg) and local infiltration anesthesia with 2% lidocaine and 1:80,000 noradrenaline. On the edentulous jaw on one side, one machined implant (diameter: 3.75 mm, length: 7 mm, Bråunemark System® MkⅢ, Sa: 0.9 μm, Nobel Biocare; Göteborg, Sweden) was placed mesially, and one anodized implant (diameter: 3.75 mm, length: 7 mm, Bråunemark System^®^ MkIII TiUniteTM, Sa: 1.1 μm, Nobel Biocare; Göteborg, Sweden) was placed distally, with a 15-mm distance between implants. On the opposite side, an anodized implant was placed mesially and a machined implant was placed distally (Figure 5).

**Figure 6:**
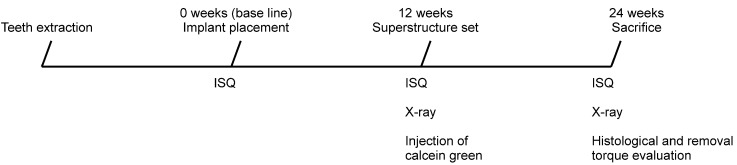
Design and time schedule of the study. ISQ, implant stability quotient.

Thus, a total of 20 implants were placed in the five dogs (10 machined implants and 10 anodized implants), and the mucosa was sutured. The time of placement was considered to be the baseline time point. The overall study design and timeline is shown in Figure 6. The timing and duration of the implantation and measurements used in the study were determined according to our previous study [9].

### 3.3. Implant Stability Evaluation

Using an Osstell^®^ (Integration Diagnostics AB, Gothenburg, Sweden) resonance frequency analyzer, we measured the implant stability quotient (ISQ) values at the time of placement and 12 and 24 weeks later. Three measurements were obtained from two directions (mesiodistally and buccolingually) and the mean value was calculated.

### 3.4. Radiographic Evaluation

Jigs were fabricated to achieve standardization of radiographs. The amount of peri-implant bone resorption was measured by digital intraoral radiograph immediately after implant placement and at 12 and 24 weeks.

**Figure 7:**
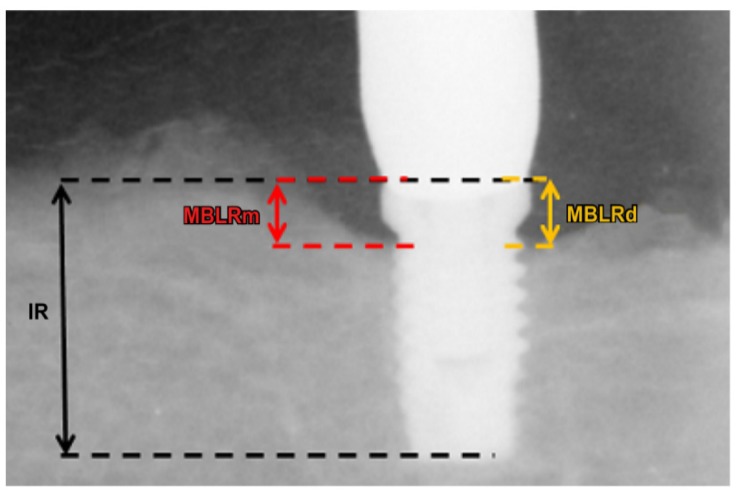
X-ray reference points for measuring vertical marginal bone loss (MBL) around the implant in the mesial aspect (MBLRm) and distal aspect (MBLRd); IR, implant length on the radiograph. Using the length of the actual implant (I), MBL was calculated from the following ratio: I/MBL = IR/MBLR.

The following indices were used to obtain the radiographic measurements (Figure 7): (1) implant length of the radiograph (IR: perpendicular distance from the implant shoulder to the most apical aspect) and (2) vertical marginal bone loss around the implant of the radiograph (MBLR: average perpendicular distance from the implant shoulder to the first visible apical bone-to-implant contact point in the mesial and distal aspects of the implant). Vertical marginal bone loss (MBL) around the implant was calculated using the length of the actual implant (I) and the following ratio: I/MBL = IR/MBLR.

The change in MBL at 12 and 24 weeks after implant placement was calculated [8]. A second surgery was performed for healing abutment installation at 10 weeks after implant placement. Two weeks later, a connecting superstructure fabricated with a gold-silver-palladium alloy (Castwel M.C., GC, Tokyo, Japan) was attached to the implants. A connecting crown was attached to the maxillary molars in order to maintain occlusal contact of the superstructures. Twelve weeks after delivered superstructure, we injected 25 mg/kg of fluorescent dye (Calcein, Sigma-Aldrich®, St. Louis, MO, USA) intravenously as an indicator of bone remodeling. The animals were fed solid food and their teeth were brushed five times a week with 0.05% chlorhexidine (Concool Gel^®^, WelTec, Osaka, Japan) to prevent mucosal inflammation around the implants or remaining teeth.

### 3.5. Removal Torque Evaluation

**Figure 8:**
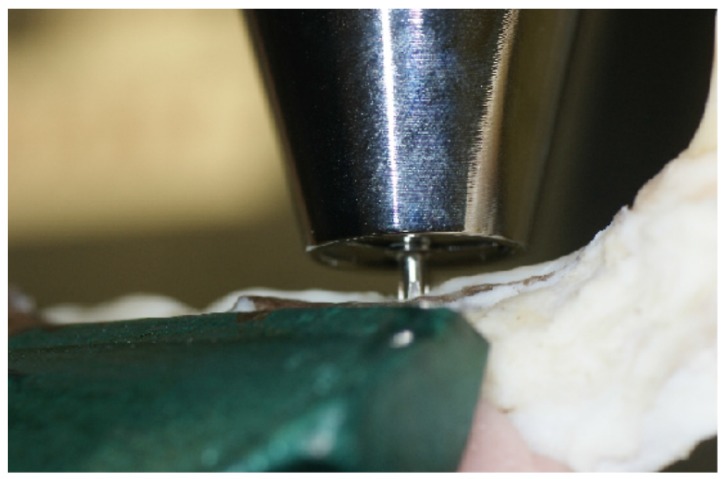
Removal torque measurement using a torque gauge.

Twenty-four weeks after implant placement, ISQ measurements and X-ray images were obtained. The animals were then killed under deep anesthesia, and bone blocks containing the implants were resected. The bone blocks with implant bodies were immediately fixed in 10% neutral formalin. The bone blocks were fixed using jigs so that the long axes of the implant bodies were perpendicular to the horizontal plane. A torque gauge with an implant driver mounted onto the tip was used to perform a counter-clockwise rotation with respect to the long axis of the implant bodies (Figure 8). The rotational force was increased until destruction of the peri-implant bone caused the implant bodies to rotate horizontally. The maximum removal torque was measured at the time of removal.

### 3.6. Histological Evaluation

The bone specimens were processed to obtain thin ground sections. Tissue blocks were dehydrated using ascending concentrations of ethanol, cleared with a styrene monomer, and then embedded in light-polymerized polyester resin (Technovit 7200VLC, Heraeus Kulzer, Germany). The resulting blocks were halved in the mesiodistal direction with respect to the implant long axis from the middle of the respective implant placement cavities (MG5000, Exakt Apparatebau, Kulzer, Germany). The specimens were sectioned with a high-precision diamond disc to produce a 200-μm-thick cross section. The non-decalcified specimens were ground to approximately 70-μm-thick sections. The sections were stained with hematoxylin-eosin and examined by light microscopy. Fluorescent observation of remodeling activity was observed using a fluorescent microscope that incorporated an epi-illumination device (BZ-9000, Keyence, Osaka, Japan).

### 3.7. Statistical Analysis

All data are presented as mean ± standard deviation. The effects of implant surface features (anodized *vs*. machined) and implant location (mesial *vs*. distal) were analyzed by two-way analysis of variance with a Tukey post-hoc test using Ekuseru-Toukei 2008 software (Social Survey Research Information Co., Ltd., Tokyo, Japan) at a significance level of 0.05.

## 4. Conclusions

The aim of this study was to clarify the implant surrounding bone on connecting superstructures with different surfaces properties. Although neither ISQ nor marginal bone loss were significantly different between the groups, removal torque of distal machined implants were significantly lower than other implants. Our limited results suggest that the most distal implants with polished surfaces need to be carefully monitored or considered.
